# Effects of Oral Lactate Supplementation on Acid–Base Balance and Prolonged High-Intensity Interval Cycling Performance

**DOI:** 10.3390/jfmk9030139

**Published:** 2024-08-20

**Authors:** Claire Bordoli, Ian Varley, Graham R. Sharpe, Michael A. Johnson, Philip J. Hennis

**Affiliations:** Sport, Health and Performance Enhancement (SHAPE) Research Centre, Nottingham Trent University, Clifton Lane, Clifton, Nottingham NG11 8NS, UK; claire.bordoli@ntu.ac.uk (C.B.); ian.varley@ntu.ac.uk (I.V.); graham.sharpe@ntu.ac.uk (G.R.S.); michael.johnson@ntu.ac.uk (M.A.J.)

**Keywords:** ergogenic aid, exercise performance, lactate supplementation

## Abstract

Lactate is an important energy intermediate and metabolic buffer, and may be ergogenic. We investigated if lactate supplementation is an effective approach to enhance the exercise performance and acid–base balance of trained cyclists during exercise devised to simulate the demands of endurance road race cycling. Sixteen endurance-trained male cyclists ($\overset{\cdot }{V}$O_2max_ 59 ± 7 mL·kg^−1^·min^−1^) consumed 120 mg·kg^−1^ body mass of lactate or a placebo 70 min prior to performing an exercise performance test, comprising five repeated blocks consisting of 1 km and 4 km time trials interspersed with 10 min of moderate-intensity exercise. Blood acid–base balance (including [H^+^] and [HCO_3_^−^]), heart rate, perceived exertion, and gastro-intestinal tolerance were assessed. There was no effect of lactate supplementation on exercise performance (*p* = 0.320), despite a reduction in RPE (*p* = 0.012) and increases in [SID] (*p* = 0.026) and [HCO_3_^−^] (*p* = 0.041). In addition, gastro-intestinal side effects were observed, but there was no effect on heart rate. Lactate supplementation did not improve exercise performance, despite positive changes in acid–base balance and RPE. This suggests that the alkalising effects of the supplement can reduce perceived effort, but these benefits do not translate into performance improvements.

## 1. Introduction

Lactate is no longer deemed a harmful waste product of glycolysis that causes muscle fatigue through associated muscle acidosis [1,2,3]. Rather, lactate is an important energy intermediate, signalling molecule, and metabolic buffer [4]. When lactate is consumed orally, it is readily absorbed into the bloodstream [5] and various tissues, including the skeletal muscle, where it can fuel oxidative metabolism [6]. Lactate can also be absorbed by hepatocytes and converted into glucose through gluconeogenesis, which can then be stored as glycogen [7]. Moreover, these reactions consume hydrogen ions, which may potentially preserve the blood bicarbonate levels and enhance the ability to buffer the extracellular pH [8,9,10]. Therefore, lactate potentially plays a multifaceted role as an ergogenic aid by supporting both energy production and the maintenance of the acid–base balance during exercise.

While lactate’s roles as an energy intermediate and metabolic buffer are well-established, its practical application as an ergogenic aid presents a more complex picture, appearing to vary with the intensity and duration of exercise. Prolonged moderate-intensity (86% max heart rate or 70% $\overset{\cdot }{V}$O_2max_) exercise tolerance is not improved with oral lactate supplementation [11,12]. Conversely, during high-intensity exercise such as sprint running or at 100% $\overset{\cdot }{V}$O_2max_, lactate supplementation has been shown to extend time to task failure by 4–26% [5,10,13], although other studies report no ergogenic effects [14,15,16,17,18]. Lactate’s ergogenic potential appears to be most significant when high-intensity exercise follows prolonged moderate-intensity activity. For example, Azevedo et al. [19] used a two-stage exercise test which consisted of participants cycling at 62% $\overset{\cdot }{V}$O_2max_ for 90 min, then at 86% $\overset{\cdot }{V}$O_2max_ until reaching their limit of tolerance. The consumption of lactate (alongside fructose, glucose, and a glucose polymer) increased time to task failure by 25% compared to consuming only fructose and glucose. Exercise tests that include long-duration, moderate-intensity exercise combined with short-duration high-intensity bouts may be most amenable to improvements through lactate supplementation, as they provide challenges to the energy metabolic pathways and buffering systems, both of which may be improved by lactates’ proposed mechanisms of action.

Road cycling is predominantly characterised by steady-state and non-steady-state exercise, in line with competitive cyclists breaking away from the pack or completing hill climbs [20,21]. The prolonged nature of road race cycling leads to the gradual depletion of muscle glycogen [22,23], thus affecting the availability of the substrates required for ATP synthesis. Additionally, high-intensity interval exercise will induce a transient metabolic acidosis, which can contribute to peripheral muscle fatigue and also central fatigue by acting on group III/IV muscle afferents and impairing overall performance [24]. Due to its mechanisms of action as an energy substrate and potential mediator of acid–base balance, lactate supplementation may offer a promising solution to mitigate the effects of these co-existing metabolic challenges on race cycling exercise performance.

Thus far, no studies have investigated the efficacy of oral lactate during a prolonged performance test that includes repeated bouts of high-intensity exercise interspersed with moderate-intensity exercise, as the nature of road race cycling can be [20,21]. Furthermore, the literature presents conflicting reports on gastro-intestinal disturbances associated with lactate supplementation [13,18,19,25], raising concerns about its suitability for prolonged exercise such as competitive cycling events. The aim of this study was to examine the effects of oral lactate supplementation on the time taken to complete a cycling exercise protocol comprising repeated bouts of high-intensity exercise interspersed with moderate-intensity exercise. Additionally, we evaluated the gastro-intestinal tolerance of oral lactate supplementation throughout the exercise protocol. It was hypothesised that the use of calcium lactate supplementation would induce significant metabolic alkalosis, leading to an enhanced cycling time trial performance.

## 2. Materials and Methods

### 2.1. Participants

Approval for the study procedures was obtained from the Nottingham Trent University Human Invasive Ethics Committee (Reference: 709) before participant recruitment. Sixteen healthy male (trained) road cyclists with at least 1 year of training history, including in prolonged (3–4 h) riding, were recruited through various means, including email circulars, posters, word of mouth, and the local cycling community. The flow of participants through the study is detailed as a CONSORT flow diagram in Supplementary File S1. All participants provided written informed consent and the research was performed in accordance with the principles stated in the Declaration of Helsinki. The participants had an average age of 26 ± 6 years, body mass of 72.2 ± 6.8 kg, height of 179 ± 5 cm, body fat of 11 ± 4%, and a $\overset{\cdot }{V}$O_2max_ of 4.3 L·min^−1^ (59 ± 7 mL·kg^−1^·min^−1^).

The G*Power software version 3.1.9.7 [26] was used to calculate the necessary sample size to ensure that our study was sufficiently powered to identify differences in the time trial performance. We estimated an effect size of 0.68, using prior research that evaluated the effects of 120 mg/kg body weight calcium lactate on exercise performance [5]. With this effect size, an alpha of 0.05, and a power setting of 0.80, it was determined that 15 participants were needed. To ensure a balanced allocation between study arms, we adjusted the sample size to 16 participants.

### 2.2. Experimental Design

A randomised, repeated-measures, double-blind crossover study was conducted. The participants visited the laboratory on four separate occasions. Visit 1 included anthropometry and a Cardio Pulmonary Exercise Test to determine their gas exchange threshold (GET) and $\overset{\cdot }{V}$O_2peak_. During visit 2, the participants completed a familiarisation trial comprising 3 of the 5 blocks of the main exercise protocol (thus, approximately 2 h of cycling). During visits 3 and 4, the participants completed the exercise protocol 70 min after consuming either calcium lactate or a placebo [5,13]. The treatment order was randomised. For visits 3 and 4, the participants arrived at the laboratory at the same time of day (0900) (1 h post-prandial) separated by at least 4 days to allow for the recovery and control of their circadian rhythms. Prior to visits 3 and 4, the participants abstained from alcohol for 24 h, caffeine for 5 h, and strenuous exercise for 48 h. The participants were requested to maintain consistent training schedules between visits and adhere to dietary restrictions, with 7-day training and 24 h food diaries collected and reviewed to assess their compliance to these conditions. Upon participant arrival, the laboratory environment’s temperature, barometric pressure, and humidity were recorded. Throughout the exercise protocol, the participants were permitted to drink water ad libitum, and their total water consumption was recorded upon completion.

### 2.3. Equipment and Measurements

**Figure 1:**
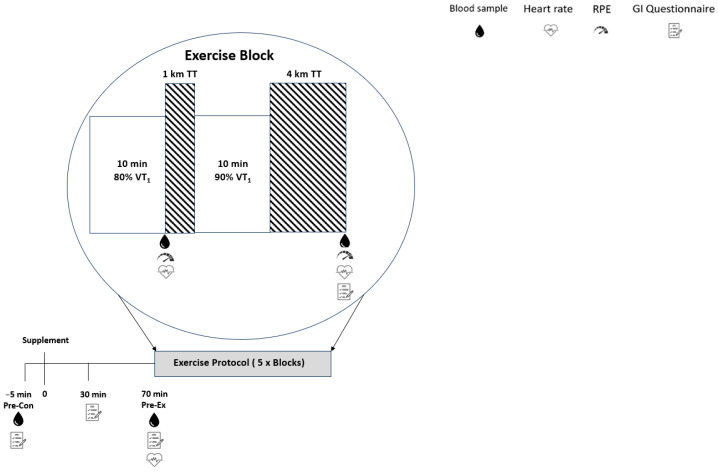
Schematic representation of the study. Pre-con: pre supplement; pre-ex: 70 min post supplement; VT_1_: ventilatory threshold; and TT: time trial.

Anthropometry was recorded, including height (to the nearest 0.01 m; Seca 217 stadiometer, Seca; Hamburg; Germany), body mass (to the nearest 0.1 kg; Seca 761 scales, Seca, Hamburg, Germany), and body composition using bioelectrical impedance (Bodystat Ltd., Isle of Man, British Isles). Exercise was performed using an electromagnetically braked cycle ergometer (Excalibur Sport; Lode, Groningen, The Netherlands). The positions of the saddle and handlebars were replicated for all trials. The participants wore a facemask (model 7940; Hans Rudolph, Kansas, MO, USA), and $\overset{\cdot }{V}$O_2peak_ and pulmonary gas exchange variables were measured breath-by-breath (Version 3B, Cortex Medical, Leipzig, Germany). Fingertip capillary blood samples were collected pre supplement consumption, pre exercise, and pre and post each exercise block (Figure 1) using 70 μL balanced heparin blood capillary tubes (Radiometer, Copenhagen, Denmark). Blood samples were immediately analysed (ABL90 Flex; Radiometer, Copenhagen, Denmark) for their concentrations of lactate [La^−^], glucose [glucose], bicarbonate [HCO_3_^−^], haemoglobin [Hb], potassium [K^+^], sodium [Na^+^], calcium [Ca^2+^], and chloride [Cl^−^], along with the pH and partial pressure of carbon dioxide PCO_2_. The hydrogen ion concentration [H^+^] was derived from the measured pH as the antilog. The strong ion difference ([SID]) was calculated as the sum of the strong ions minus the sum of the strong anions: [SID] = ([Na^+^] + [K^+^] + [Ca^2+^]) − ([Cl^−^] + [La^−^]) [27,28]. Changes in the blood volume from baseline were calculated from the changes in [Hb] [29]. Heart rate was measured continuously via telemetry (Sigma ID. GO, Sigma-electro, Neustadt, Germany). Ratings of perceived exertion (RPEs) were recorded using the Borg scale (6–20) [30], immediately before (pre) and within the last few seconds (post) of each exercise block. Gastro-intestinal tolerance was assessed using a questionnaire based on previous studies [31,32]. The participants rated their level of gastro-intestinal intolerance using a Likert scale, assessing the symptoms of nausea, flatulence, stomach cramping, belching, stomach ache, bowel urgency, diarrhoea, vomiting, and stomach bloating. The participants indicated the severity of each symptom on a scale from 0 to 10, with 0 being “no symptom” and 10 being “severe symptom”. These severity ratings were recorded pre supplement consumption, 30 min post supplement consumption, immediately pre-exercise, and immediately after each exercise block. The time to complete the exercise protocol, which served as the primary outcome of the study, was recorded using the Lode Ergometry Manager 10 Software (Lode, Groningen, Germany).

### 2.4. Cardio Pulmonary Exercise Test (CPET)

The participants performed 3 min of rest and 3 min of unloaded cycling, followed by an incremental ramp protocol (35 W·min^−1^ or 40 W·min^−1^) at their preferred cadence until task failure (cadence below 60 rpm, despite verbal encouragement). The $\overset{\cdot }{V}$O_2peak_ was defined as the average of the exertional oxygen uptake achieved over the last 30 s of exercise. The GET was determined using the modified V-slope method [33], confirmed by patterns of change in the ventilatory equivalent and end-tidal gas measurements, later verified by an independent researcher. The power output at the GET was adjusted by subtracting two-thirds of the ramp increment per minute, i.e., the power output—0.67 × ramp increment, in order to convert the ramp exercise to a steady state. The GET was then used to determine the intensity of the steady-state exercise (80% and 90% GET) within the main exercise trial.

### 2.5. Supplementation Strategy

The participants consumed 147 mg·g^−1^ body mass of either calcium lactate (Special Ingredients Ltd., Chesterfield, UK) or a placebo (flour) within opaque gelatine capsules over a 5–10 min period 70 min before exercise. This dosage was selected to provide 120 mg·kg^−1^ body mass of lactate [5,13]. The calcium lactate used was obtained from a factory-sealed container (Special Ingredients Ltd., Chesterfield, UK) and was weighed to the nearest milligram before being placed in the capsules. The number and colour of the capsules were consistent across treatments. The selection of the calcium lactate dosage was based on previous studies, where significant increases in peak blood [HCO_3_^−^] between pre and post consumption were observed (26.83 ± 2.53 vs. 29.50 ± 1.96 mmol^−1^ [13]; 29 ± 2.9 vs. 32.0 ± 1.6 mmol·L^−1^ [5], with no further increases at higher doses.

### 2.6. Dietary Control

The participants were provided with a standardised evening meal and standardised breakfast prior to visits 3 and 4. The evening meal, consumed at 1900 h the night before visits 3 and 4, provided 40% of the participants’ estimated energy requirements, with a specific macronutrient composition based upon 50% carbohydrates, 35% fat, and 15% protein [34]. The breakfast, consumed at 0800 h, provided 2 g of carbohydrates per kg of body mass [35,36].

### 2.7. Exercise Protocol

The exercise protocol comprised 5 repeated exercise blocks. Each block consisted of 10 min of cycling at 80% GET followed by a 1 km TT, then 10 min at 90% GET followed by a 4 km TT. During the TTs, resistance to pedalling was set using the linear mode of the cycle ergometer, in which the power output was dependent on the cycling cadence. The linear factor for the 1 km TT and 4 km TT was calculated based on specific reductions in the absolute peak power during 1 km and 4 km TTs observed in previous research [35,37], and the participants’ preferred cadence following familiarisation. Before each TT, the investigator provided a countdown, instructed the cyclists to complete the TT as quickly as possible, and provided verbal encouragement throughout. During the time trials, the participants were blinded to all performance data, except for the distance countdown.

### 2.8. Statistical Analysis

The data analysis was conducted using SPSS statistical software version 28.0 (IBM Corporation, Armonk, NY, USA). Initially, the accuracy of the data entry and the presence of missing values were examined. Some data points (n = 16) were missing completely at random due to machine or human errors, and an expectation maximisation imputation method was used to replace these missing values. In some instances, data were systematically missing due to machine malfunctions, which affected the blood biochemistry data (n = 2), and due to human error, which affected the heart rate and perceived exertion data (n = 3), leading to the exclusion of these participant data. The normal distribution of the data was assessed using the Shapiro–Wilk test, and appropriate inferential statistics were conducted based on the results. Student paired *t*-tests were used to evaluate the differences between treatments (calcium lactate and placebo supplement) in delta changes in the blood measures pre and post supplement consumption, environmental laboratory conditions, and total water consumption during the exercise. A two-way (Treatment × Time) repeated-measures analysis of variance (ANOVA) was used to identify the differences in the time to complete each 1 km TT and each 4 km TT. Three-way (Treatment: 2 levels: calcium lactate, placebo) × Block (5 levels: exercise block 1 to 5) × Time (2 levels: pre and post each exercise block) ANOVAs were used to identify the differences in the concentrations of blood [La^−^], [glucose], [H^+^], [HCO_3_^−^], [Hb], PCO_2_, [K^+^], [Na^+^], [Ca^2+^], [Cl^−^], and [SID], heart rate, and RPE. The homogeneity of variance was assessed using the Mauchly test, and in cases where the assumption of sphericity was violated, a Greenhouse–Geisser correction was applied. The effect sizes are reported using partial eta squared (η^2^_p_), with magnitudes of small (η^2^_p_ = 0.01), medium (η^2^_p_ = 0.06), and large (η^2^_p_ = 0.14) [38]. For the student paired *t*-tests, the effect sizes are presented as Cohen d_z_ and interpreted as small (d_z_ = 0.2), medium (d_z_ = 0.5), and large (d_z_ = 0.8) [38]. Statistical significance was set at *p* < 0.05. Data are presented as means with 95% confidence intervals. To examine any differences in gastro-intestinal symptoms over time for each treatment, a Friedman’s test was performed. The effect sizes are presented as Kendall’s W (Kendall coefficient of concordance) and interpreted as small (W = 0.1), medium (W = 0.3), and large (W = 0.5) [38]. Pairwise comparisons of gastro-intestinal symptoms following each exercise block between treatments were then conducted using the Wilcoxon signed-rank test. The effect sizes are interpreted as small (r = 0.1), medium (r = 0.3), and large (r = 0.5) [38]. Statistical significance was set at *p* < 0.05.

## 3. Results

### 3.1. Acid–Base Balance and Metabolic Measurements Pre and Post Supplementation

Table 1 presents the changes in the acid–base and metabolic measurements from pre to post supplementation for both the calcium lactate and placebo treatments. Significant differences in the pre–post deltas were observed between the calcium lactate and placebo supplementation for [SID] (*p* = 0.032, dz = 0.640), [HCO_3_^−^] (*p* = 0.019, dz = 0.714), and [Cl^−^] (*p* = 0.006, dz = −0.868). Specifically, there was a significant increase in [SID] and [HCO_3_^−^] and a significant decrease in [Cl^−^] following calcium lactate supplementation compared to the placebo. Changes in other acid–base and metabolic measures were not significantly different between the treatment groups (*p* > 0.05).

### 3.2. Performance Data

There was no main effect of Treatment on the time to complete each exercise block, indicating no change in the overall total time to complete the exercise protocol (calcium lactate: 133.60 min [130.28, 136.91]; placebo: 134.06 min [130.73, 137.36], *p* = 0.321, d_z_ = 0.257).

Additionally, there were no interaction effects of Treatment × Time on the time to complete each 1 km TT (*p* = 0.708, η^2^_p_ = 0.019) or on the time to complete each 4 km TT (*p* = 0.275, η^2^_p_ = 0.082) (Supplementary File S2). Furthermore, there were no differences in the laboratory environment’s temperature, barometric pressure, and humidity between the treatments (all *p* > 0.05). In addition, the total water consumption during exercise did not differ between the treatments (*p* = 0.895).

### 3.3. Heart Rate and Perceived Exertion

**Figure 2:**
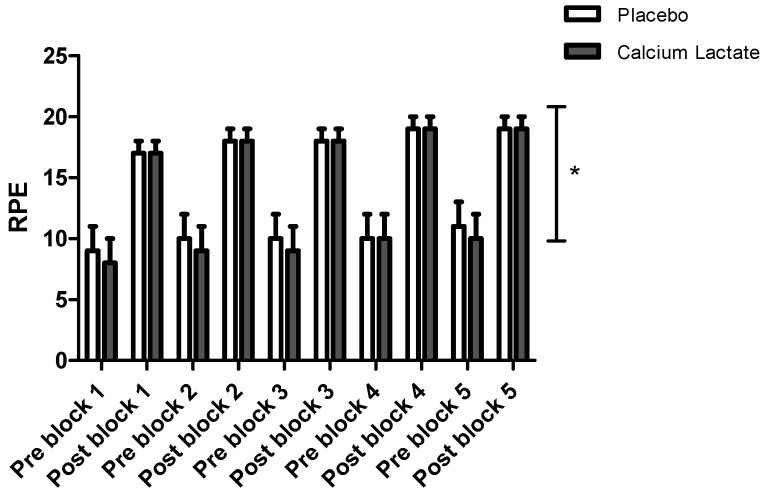
Ratings of perceived exertion pre and post each exercise block (n = 13). RPE = ratings of perceived exertion. Results are expressed as mean (95% confidence interval). * Indicates significant main effect of Treatment (* *p* < 0.05).

There was no main effect of Treatment (*p* = 0.536, η^2^_p_ = 0.033) or an interaction effect of Treatment × Block × Time on heart rate (*p* = 0.298, η^2^_p_ = 0.095). In contrast, there was a significant main effect of Treatment on RPE (Figure 2), with a significant reduction in RPE with calcium lactate supplementation (*p* = 0.012, η^2^_p_ = 0.423); however, no interaction effects were observed (all *p* > 0.05).

### 3.4. Acid–Base Balance and Metabolic Measurements during Exercise

**Figure 3:**
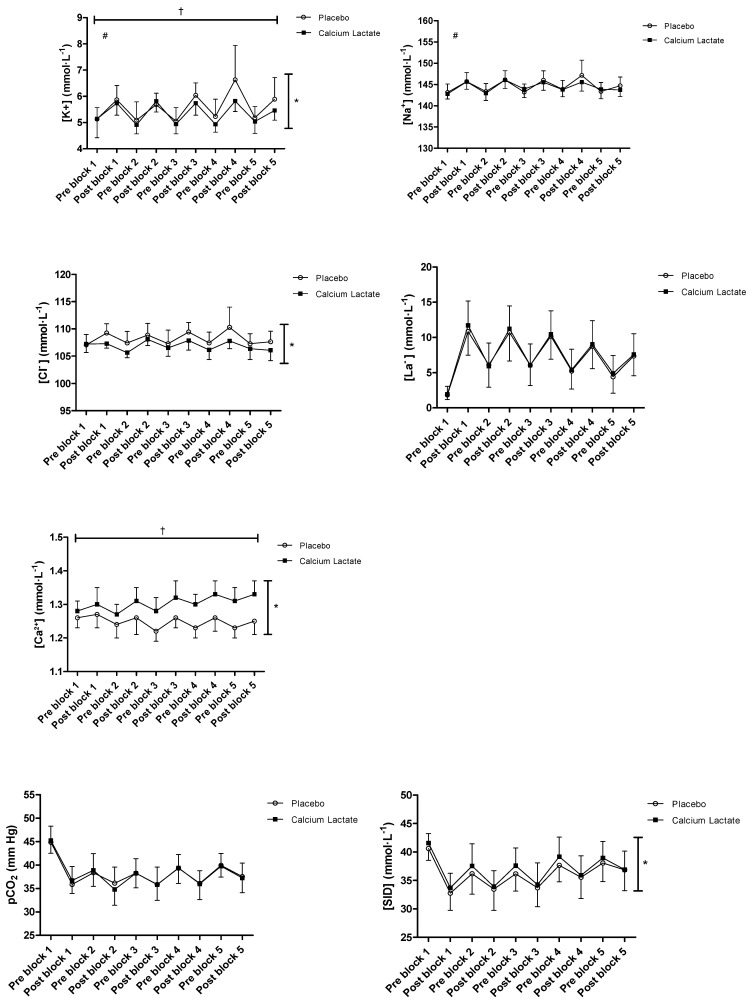

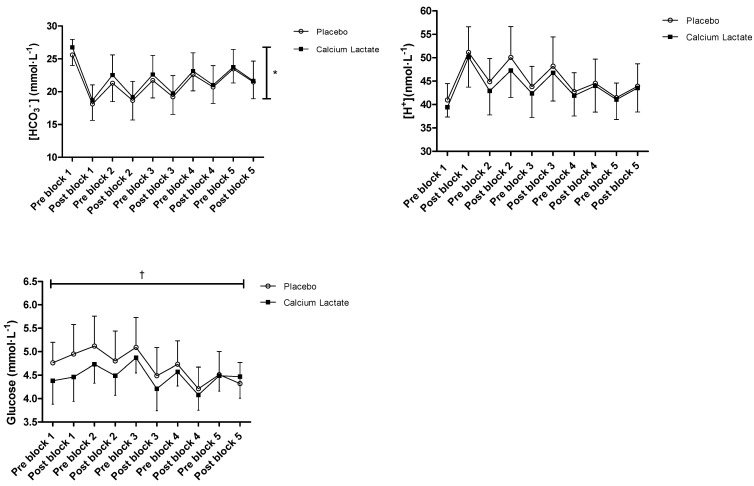
Blood acid–base balance and glucose pre and post each exercise block (n = 14). Results are expressed as means (95% confidence interval). * Indicates significant main effect of Treatment (* *p* < 0.05). ^†^ Indicates significant Treatment × Block effect (* *p* < 0.05). ^#^ Indicates significant Treatment × Time effect (* *p* < 0.05).

There was a main effect of Treatment on [K^+^] (4% lower in calcium lactate than placebo) (*p* = 0.002, η^2^_p_ = 0.528) (Figure 3). Interaction effects of Treatment × Block (*p* = 0.018, η^2^_p_ = 0.201) and Treatment × Time (*p* = 0.040, η^2^_p_ = 0.285) were noted, though no three-way interaction effect was found. There was no main effect of Treatment on [Na^+^] (*p* = 0.298, η^2^_p_ = 0.083), but there was a Treatment × Time interaction effect (*p* = 0.016, η^2^_p_ = 0.370). There was a main effect of Treatment on [Cl^−^], with calcium lactate supplementation leading to lower [Cl^−^] (*p* < 0.001, η^2^_p_ = 0.598), however, no Treatment interaction effects were observed (all *p* > 0.05). There was no main effect of Treatment on [La^−^] (*p* = 0.348, η^2^_p_ = 0.068) or interaction effects of Treatment × Block × Time (*p* = 0.615, η^2^_p_ = 0.040). There was a main effect of Treatment on [Ca^2+^], with calcium lactate supplementation leading to higher [Ca^2+^] (*p* < 0.001, η^2^_p_ = 0.855). There was also a Treatment × Block interaction effect (*p* < 0.001, η^2^_p_ = 0.619), however, no Treatment × Block × Time interaction effect was observed (*p* = 0.605, η^2^_p_ = 0.44).

There was no significant main effect or interaction effects of Treatment on pCO_2_ (all *p* > 0.05). There was a main effect of Treatment on [SID], with increased [SID] occurring with calcium lactate supplementation (*p* = 0.026, η^2^_p_ = 0.327). There was a main effect of Treatment on [HCO_3_^−^], with calcium lactate supplementation leading to higher [HCO_3_^−^] (*p* = 0.041, η^2^_p_ = 0.284). There was no main effect of Treatment on [H^+^] (*p* = 0.056), although the effect size was large (η^2^_p_ = 0.254). For [HCO_3_^−^], [SID], and [H^+^], there were no Treatment × Block × Time interaction effects (*p* > 0.05).

There was no main effect of Treatment on [Glucose] (*p* = 0.097, η^2^_p_ = 0.197) and no Treatment × Block × Time interaction effect (*p* = 0.642, η^2^_p_ = 0.046). There was, however, a Treatment × Block interaction effect (*p* = 0.028, η^2^_p_ = 0.246), with less of a decline in [Glucose] observed in the calcium lactate group compared to the placebo group over the course of the exercise blocks.

### 3.5. Gastro-Intestinal Tolerance

Flatulence (*p* = 0.027, W = 0.171), stomach cramps (*p* = 0.046, W = 0.151), belching (*p* = 0.022, W = 0.179), stomach ache (*p* = 0.020, W = 0.182), and bowel urgency (*p* = 0.035, W = 0.161) were all found to increase significantly during the exercise protocol following lactate supplementation, whereas no significant changes were found in nausea (*p* = 0.103, W = 0.120), diarrhoea (*p* = 0.406, W = 0.063), vomiting (*p* = 0.607, W = 0.042), or stomach bloating (*p* = 0.115, W = 0.116). In contrast, no significant increases in any symptoms were found during the exercise protocol following consumption of the placebo (all *p* > 0.05). Pairwise comparisons between interventions at individual time points only showed that, compared to the placebo, bowel urgency was significantly higher after calcium lactate supplementation following the third 4 km TT (z = 2.060, r = 0.515, *p* = 0.039).

## 4. Discussion

This study represents the first investigation into the effects of oral lactate supplementation on exercise performance during prolonged exercise that simulated the demands of endurance road race cycling. It was hypothesised that this specific type of activity, given lactate’s proposed buffering capacity [5,8,10,13,18,39] and potential impact on energy availability [19], may be particularly responsive to improvements. Our findings demonstrated that lactate supplementation positively influenced acid–base balance, with significant elevations in [HCO_3_^−^] and [SID], along with a reduction in perceived exertion. However, despite these changes, there were no discernible effects on cycling time trial performance. These results are in agreement with others that have shown increased blood [HCO_3_^−^] following lactate supplementation [5,12,13,14,18,39] and reductions in perceived exertion [40]. However, in contrast to others [5,10,13], lactate supplementation in the present study failed to yield exercise performance benefits and, consequently, our study provides evidence that challenges the efficacy of lactate supplementation as an ergogenic aid, particularly in the context of prolonged intermittent exercise.

Acute calcium lactate supplementation decreased the resting blood [Cl^−^] by 2 mmol·L^−1^. Since other ions were unchanged at rest, the increased [Cl^−^], therefore, accounted for the approximately equimolar increase in [SID] and, subsequently, the increase in [HCO_3_^−^]. Compared to the placebo, calcium lactate supplementation also resulted in a mean [SID] that was 0.74 mmol·L^−1^ higher during exercise. Since there were no differences in pCO_2_ between treatments, the higher [SID] can largely account for the lower [H^+^] (evidenced by the large effect size) and higher [HCO_3_^−^] after the calcium lactate supplementation [28], although we cannot discount a slight role played by between-trial differences in the total weak acid concentration [28]. The higher [SID] during exercise after calcium lactate supplementation resulted from the net effect of changes in several strong ions. Specifically, while the lower [K^+^] after calcium lactate supplementation would, by itself, reduce [SID], this was numerically offset by the higher [Ca^2+^] and (to a greater extent) the lower [Cl^−^], which would both increase [SID]. The mechanisms that explain why the calcium lactate supplementation resulted in lower [Cl^−^] at rest and, compared to the placebo, lower [Cl^−^] and [K^+^] during exercise, are beyond the scope of the present study, but could be related to differences in water shifts and, therefore, plasma volume [41,42] and/or alterations in ion transport systems [43]. Reduced acid–base perturbation during exercise after calcium lactate supplementation may also influence the ensemble group III/IV muscle afferent feedback [24], which may explain, in part, the reduced RPE observed in the present study [44,45].

Previous studies have demonstrated positive responses in acid–base balance following acute lactate supplementation. Morris et al. [5,13] demonstrated, in two separate studies, improvements in acid–base balance using the same lactate dose and supplementation strategy as the current study, with significant increases in blood [HCO_3_^−^], but no significant changes in pH. However, in contrast, they observed significant improvements in intermittent exercise. Considering the evidence from Morris et al. [5,13], it was reasonable to expect that, within the current study, the same dose of 120 mg·kg^−1^ body mass, administered 70 min before exercise, would enhance performance, particularly as higher doses have not been associated with additional improvements [5,18] and the buffering effects of lactate are sustained for 140 min post-administration [13]. There are, however, several differences between our work and that of Morris et al. [5,13], which may have contributed to the differences shown in the performance effects. A notable difference was that the current study showed an increase in blood [HCO_3_^−^] of 4% (25.76 to 26.68 mmol·L^−1^) between pre and post supplementation, which is lower than the 10% increase observed by Morris et al. [5,13]. The reasons that our study did not observe a similar increase in [HCO_3_^−^] following supplementation are unclear, but may explain the lack of performance effects. Furthermore, the nature of the exercise within the current study was significantly different compared to that of Morris et al. [5,13], who evaluated the time to exhaustion following multiple bouts of high-intensity exercise of a significantly shorter duration. This type of exercise significantly increases acidosis, eliciting twice the increase in [H^+^] levels [46] compared to our current findings. Consequently, it might be more responsive to the alkalising effects of lactate, which could lead to an enhanced performance. Furthermore, it appears clear that these differences in the exercise protocols likely led to the varying incidence of gastro-intestinal symptoms reported. The ratings of perceived illness and stomach ache were low and insignificant, with no reports of gastro-intestinal symptoms affecting performance in the work of Morris et al. [5,13]. This is not surprising given the significantly shorter duration of exercise within their studies and that, within the current study, gastro-intestinal symptoms such as flatulence, stomach cramps, belching, stomach ache, and bowel urgency all increased as the exercise progressed, thus potentially impacting performance. Overall, the discrepancies between our study and the findings from Morris et al. [5,13] indicate that the changes in acid–base perturbation and RPE after the calcium lactate supplementation in the present study were not sufficiently large enough to elicit an improvement in exercise performance. This suggests that the dosing strategy, including the form, amount, and frequency of lactate supplementation for performance improvement, requires further investigation [47], particularly for this type of exercise protocol.

### Strengths and Limitations

Our study has several strengths, including the randomised double-blind crossover design that allowed for the effects of calcium lactate to be directly compared against a placebo. Within the study, we implemented strict dietary control prior to exercise, with the provision of standardised meals and strict dietary restrictions in order to minimise any nutritional influence on metabolism and, subsequently, exercise performance. Furthermore, the study has a high external validity, as the exercise protocol included is reflective of the intensities found within competitive cycling endurance events with bouts of moderate-intensity exercise interspersed with high-intensity exercise, mimicking competitive road race cycling. As such, it is applicable to real-life competitive endurance events [20,21]. There are several limitations in the present study that future research should address. Future studies exploring lactate’s efficacy should consider using sodium lactate instead of calcium lactate. This change could potentially enhance the supplement’s effects, as sodium lactate might support greater lactate uptake through sodium-coupled monocarboxylate transporters [48], and sodium also plays a direct role in increasing the strong ion difference ([SID]). Moreover, considering the duration of our trial (approximately 2 h), it might have been beneficial to administer multiple doses of lactate to sustain the changes in the acid–base balance required to evoke a performance effect. However, studies evaluating the performance effects of frequent smaller doses of lactate are less convincing compared to studies using single higher doses (120 mg·kg^−1^ body mass and above) [5,10,11,13,39]. Future studies might, therefore, consider incorporating a single high dose prior to exercise followed by additional multiple doses in order to elicit greater and more sustained changes in acid–base balance and perceived exertion, which may then translate to an improved performance during prolonged exercise. However, this may prove difficult to implement due to the negative effect that this may have on gastro-intestinal symptoms. In addition, unlike others [19,25], we did not assess the lactates oxidation rate throughout exercise via isotope tracers and, therefore, could not assess the metabolism of lactate, which may have increased our understanding as to why there was no effect on performance.

## 5. Conclusions

The current study found no improvements in prolonged high-intensity interval cycling time trial performance following calcium lactate supplementation, despite reduced perceived exertion and changes in acid–base perturbation. These findings, in comparison to other studies, indicate that the dosing strategy utilised may have been insufficient to elicit the required influence on acid–base balance for performance enhancement, specifically for exercise of this intensity and duration. Future studies need to consider the optimal dose and form of lactate that can be practically administered, minimising potential gastro-intestinal side effects while maximising cycling time trial performance benefits.

## Figures and Tables

**Table 1:** Acid–base balance and metabolic measurements pre and post supplementation (n = 14).

	Pre Supplementation	Post Supplementation	Δ
Treatment			
Calcium Lactate			
[K^+^] mmol·^−1^	4.91 (4.62, 5.20)	4.81 (4.62, 5.00)	−0.10 (−0.38, 0.18)
[Na^+^] mmol·L^−1^	143 (142, 143)	142 (141, 143)	−0.57 (−1.52, 0.38)
[Cl^−^] mmol·L^−1^	107 (106, 108)	105 (105, 106)	−2.07 (−2.77, −1.37) *
[La^−^] mmol·L^−1^	2.14 (1.78, 2.50)	1.86 (1.63, 2.10)	−0.27 (−0.59, 0.05)
[Ca^2+^] mmol·L^−1^	1.24 (1.22, 1.26)	1.26 (1.25, 1.28)	0.02 (0.01, 0.03)
pCO_2_ mm Hg	41.31 (39.84, 42.77)	42.68 (41.60, 43.76)	1.37 (0.01, 2.73)
[SID] mmol·L^−1^	39.44 (38.61, 40.28)	41.14 (40.12, 42.15)	1.69 (0.29, 3.10) *
[HCO_3_^−^] mmol·L^−1^	25.79 (25.11, 26.46)	26.68 (26.14, 27.22)	0.89 (0.07, 1.71) *
[H^+^] nmol·L^−1^	38.52 (37.29, 39.75)	38.06 (36.98, 39.14)	−0.46 (−1.50, 0.59)
[Glucose] mmol·L^−1^	5.11 (4.80, 5.43)	5.11 (4.77, 5.46)	0.00 (−0.48, 0.48)
Placebo			
[K^+^] mmol·L^−1^	4.94 (4.60, 5.27)	4.97 (4.76, 5.18)	0.03 (−0.40, 0.47)
[Na^+^] mmol·L^−1^	143 (142, 144)	142 (141, 143)	−1.00 (−1.78, −0.22)
[Cl^−^] mmol·L^−1^	107 (106, 108)	106 (105, 107)	−0.64 (−1.42, 0.14)
[La^−^] mmol·L^−1^	2.08 (1.76, 2.40)	1.73 (1.42, 2.05)	−0.34 (−0.73, 0.04)
[Ca^2+^] mmol·L^−1^	1.25 (1.23, 1.26)	1.25 (1.24, 1.26)	0.00 (−0.01, 0.02)
pCO_2_ mm Hg	41.88 (40.91, 42.86)	42.70 (41.40, 44.00)	0.82 (−0.45, 2.08)
[SID] mmol·L^−1^	40.19 (39.16, 41.21)	40.20 (39.21, 41.20)	0.02 (−0.94, 0.97)
[HCO_3_^−^] mmol·L^−1^	25.95 (25.40, 26.49)	25.76 (25.21, 26.32)	−0.18 (−0.74, 0.38)
[H^+^] nmol·L^−1^	38.62 (37.80, 39.44)	39.30 (38.21, 40.39)	0.68 (−0.51, 1.88)
[Glucose] mmol·L^−1^	5.62 (5.27, 5.97)	5.29 (5.01, 5.58)	−0.32 (−0.86, 0.21)

Results are expressed as mean (95% confidence interval). Differences in the delta (pre to post supplementation changes) between treatments are indicated by * *p* < 0.05.

## Data Availability

Please contact the corresponding author for access.

## Supplementary Materials

The following supporting information can be downloaded at: https://www.mdpi.com/article/10.3390/jfmk9030139/s1.
