# Supplementary material for: Effects of Oral Lactate Supplementation on Acid–Base Balance and Prolonged High-Intensity Interval Cycling Performance
Source: J Funct Morphol Kinesiol. 2024 Aug 20;9(3):139. doi: 10.3390/jfmk9030139 (PMC11348031; doi:10.3390/jfmk9030139)
Supplement: Supplementary file 1 [file jfmk-09-00139-s001.zip › SupplementaryFile_2_1kmTT4kmTT.pdf]

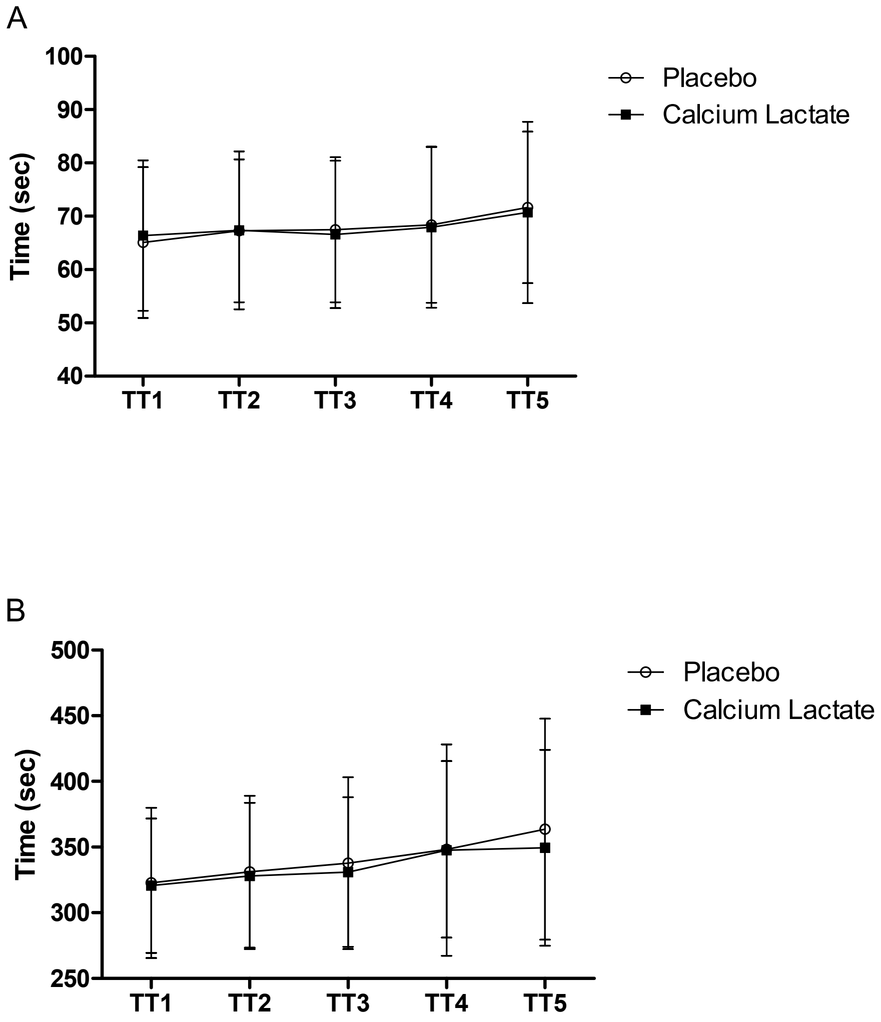

Supplementary File 2. Time to complete each 1 km time trial (A) and time to complete each 4km time trial (B). TT= Time trial. Results are expressed as mean (SD)
